# ESTs from the microsporidian *Edhazardia aedis*

**DOI:** 10.1186/1471-2164-9-296

**Published:** 2008-06-20

**Authors:** Erin E Gill, James J Becnel, Naomi M Fast

**Affiliations:** 1 Department of Botany, University of British Columbia, Vancouver, BC, V6T 1Z4, Canada; 2 Center for Medical, Agricultural and Veterinary Entomology, USDA/ARS, Gainesville, FL 32608, USA

## Abstract

**Background:**

Microsporidia are a group of parasites related to fungi that infect a wide variety of animals and have gained recognition from the medical community in the past 20 years due to their ability to infect immuno-compromised humans. Microsporidian genomes range in size from 2.3 to 19.5 Mbp, but almost all of our knowledge comes from species that have small genomes (primarily from the human parasite *Encephalitozoon cuniculi *and the locust parasite *Antonospora locustae*). We have conducted an EST survey of the mosquito parasite *Edhazardia aedis*, which has an estimated genome size several times that of more well-studied species. The only other microsporidian EST project is from *A. locustae*, and serves as a basis for comparison with *E. aedis*.

**Results:**

The spore transcriptomes of *A. locustae *and *E. aedis *were compared and the numbers of unique transcripts that belong to each COG (Clusters of Orthologous Groups of proteins) category differ by at most 5%. The transcripts themselves have widely varying start sites and encode a number of proteins that have not been found in other microsporidia examined to date. However, *E. aedis *seems to lack the multi-gene transcripts present in *A. locustae *and *E. cuniculi*. We also present the first documented case of transcription of a transposable element in microsporidia.

**Conclusion:**

Although *E. aedis *and *A. locustae *are distantly related, have very disparate life cycles and contain genomes estimated to be vastly different sizes, their patterns of transcription are similar. The architecture of the ancestral microsporidian genome is unknown, but the presence of genes in *E. aedis *that have not been found in other microsporidia suggests that extreme genome reduction and compaction is lineage specific and not typical of all microsporidia.

## Background

Microsporidia are single-celled eukaryotic intracellular parasites that are related to fungi. Currently, over 1200 species have been identified, infecting animals from nearly every phylum, including commercially important species such as honeybees and fish, as well as humans [1]. Inside host cells, microsporidia proliferate as vegetative stages (meronts, schizonts) which eventually produce spores that are released when the host cell lyses. Spores possess a unique host cell invasion apparatus called the polar filament, which is forcefully everted upon germination to form a tube and can pierce a nearby host cell [1]. The tube then acts as a conduit allowing the contents of the spore to be injected into the host cell's cytoplasm, where the parasite undergoes vegetative replication.

Microsporidia are a diverse group of organisms, and vary greatly in the complexity of their life cycles. For instance, *Encephalitozoon cuniculi *and *Antonospora locustae *produce only one type of spore (uninucleate in the former and binucleate in the latter), and complete their entire life cycles inside one host individual, while *Amblyospora californica *requires two host groups (mosquitoes and microcrustacea) and produces three morphologically and functionally discrete spore types [1].

**Figure 1:**
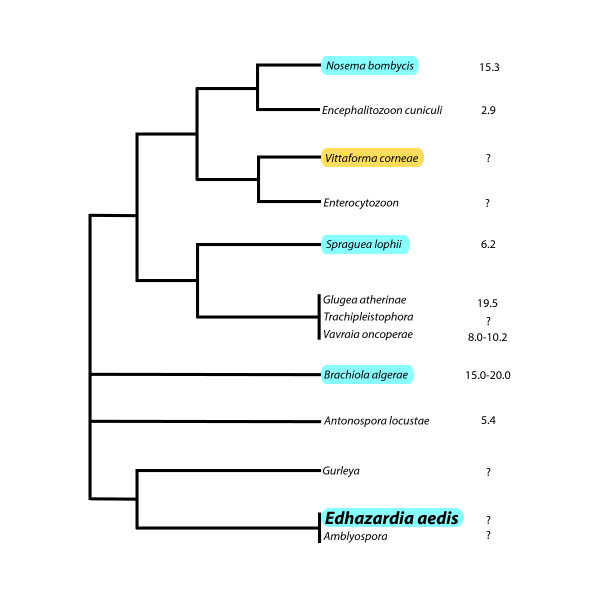
**The phylogenetic relationships between several microsporidia.** Species that house transposable elements belonging to the Ty3/gypsy family are highlighted in blue, while species containing LTR transposons are highlighted in yellow. Genome sizes are indicated to the right of each species. (Adapted from Slamovits et al., 2004.)

Microsporida possess some of the smallest primary nuclear genomes known (as tiny as 2.3 Mbp). The only microsporidian whose genome has been completely sequenced is the human parasite, *E. cuniculi*. At a meager 2.9 Mbp, *E. cuniculi*'s genome is extremely compact, with only 2000 genes [2]. A small genome sequence survey (GSS) project has been conducted on *A. locustae*, a locust parasite that has been approved as a biological control agent in the United States [3]. *A. locustae*'s genome is roughly 5.4 Mbp in size [4], or about twice the size of *E. cuniculi*'s genome. Despite the genome size difference, both genomes appear to be structured in much the same way. Genes are closely packed (nearly one gene per kilobase), are small in size compared to homologues in animals and fungi, and are intron-poor. There is also a much greater degree of synteny between these two organisms than would be expected given their phylogenetic relationship, which implies that although microsporidian genes are fast-evolving, genomic rearrangements occur only rarely [3] (See Fig. 1).

However, we have very little information on microsporidian genomes of larger sizes. *Edhazardia aedis *is a microsporidian that infects *Aedes aegypti*, the mosquito vector of the Dengue hemorrhagic and yellow fever viruses. *E. aedis *has been intensively studied as a viable biological control agent for *A. aegypti *[5] and has a genome estimated to be to be many times larger than that of *E. cuniculi*. There are several possible explanations for this difference: *E. aedis *may have more genes that control its complex life cycle. Genes may also be longer, more widely spaced, and contain more introns than *E. cuniculi *[2].

Morphological studies conducted on *E. aedis *have revealed at least four different types of spores – two uninucleate and two binucleate [6,7]. The two types of uninucleate spore types differ morphologically but possess similar pyriform shapes. However, the cell division events from which they arise differ. Spores produced via mitosis are roughly 8.5 μm in length, whereas spores produced via meiosis (meiospores) are about 7.5 μm. Small binucleate spores (~6.5 μm in length) that have short polar filaments are formed first, followed by the production of larger binucleate spores (~9 μm in length) that are ovoid in shape. Meiospore formation is usually abortive and rarely produces normal spores [6].

*E. aedis' *life cycle is moderately complex and involves two generations of the mosquito host. It begins when a uninucleate spore is ingested by a mosquito larva from the environment. Once in the gut, the spore germinates and begins to multiply in the host tissue. Within 48 hours, small binucleate spores are formed that are responsible for spread to other tissues. Orally infected larvae generally exhibit reduced growth, and may die before reaching maturity if the parasite load is high, thus releasing more spores into the environment. However, if the infection load is sufficiently small, the larva will mature into an adult mosquito and survive to reproduce [6]. If the adult mosquito is female, large binucleate spores will develop in her ovaries and will infect oocytes, thus passing the infection on to the next generation where the majority of mortality occurs in larvae. Little is known about the factors that modulate the transition from one phase in the life cycle to the next, or about the changes in gene expression that occur during these transitions.

It is also possible that the difference in genome size between *E. aedis *and *E. cuniculi *or *A. locustae *may have less to do with the number of genes, and more to do with genome architecture. *E. aedis *genes could be longer, more widely spaced, and contain more introns than *E. cuniculi *[2]. In an effort to learn more about *E. aedis*' genome, a GSS of >200 kbp was conducted [8]. This study concluded that *E. aedis*' genome structure is very different from those of *E. cuniculi *and *A. locustae*. A large portion of the genome is occupied by non-coding DNA and genes are not closely packed together, although the existence of local areas of compaction could not be ruled out.

Previous examinations of ESTs from microsporidia have only been conducted on microsporidia with small genomes. These transcripts possessed unusual features that are atypical in eukaryotes. Examinations of ESTs from *A. locustae *[9] and transcripts from *E. cuniculi *revealed numerous multi-gene transcripts. These transcripts are different from prokaryotic operons, as the proteins encoded by the transcript do not have related functions and are often not encoded on the same DNA strand. Many transcripts encode only a portion of one gene, while the other is present in its entirety [9,10]. The reason for this phenomenon is not known, but it has been suggested that transcriptional control elements have been lost (or moved into adjacent genes) during the process of genome compaction [10].

As *E. aedis*' genome and life cycle are very different from *E. cuniculi *and *A. locustae*, it is reasonable to assume that the transcript structure and number of genes present may differ as well. In this study, we describe the first survey of ESTs from a microsporidian with a much larger genome size and complex life cycle. In sequencing over 1300 transcripts, we have elucidated more of *E. aedis*' genome content, and have gained a profile of its transcript structure and composition. Surprisingly, the *E. aedis *uninucleate spore transcriptome is remarkably similar to that of *A. locustae*.

## Results

### Overview

Sequences were deposited into the Genbank EST database and have the accession numbers FG063843 to FG065106. From the 1307 clones sequenced, 133 unique genes were found; 55 were represented by a single transcript, while the remaining 78 were represented by two or more. 97 of the 133 unique genes are present in other microsporidia (See Table 1), while 10 are present in other (non-microsporidian) organisms (See Table 2), 18 are putatively *E. aedis*-specific and 8 have no apparent open reading frames. Coding sequences contained 43% G+C while 5' and 3' untranslated regions possessed 27% and 26%, respectively.

**Table 1:** 

**Gene Name**	**Species**	**Genbank Accession Number**
16S rRNA GENE	*Brachiola algerae*	AM422905
1-ACYL-SN-GLYCEROL-3-PHOSPHATE ACYLTRANSFERASE	*Encephalitozoon cuniculi*	NP_586146
26S PROTEASOME REGULATORY SUBUNIT 4	*Encephalitozoon cuniculi*	NP_586091
26S PROTEASOME REGULATORY SUBUNIT 6	*Encephalitozoon cuniculi*	NP_586128
26S PROTEASOME REGULATORY SUBUNIT 8	*Encephalitozoon cuniculi*	XP_955738
40S RIBOSOMAL PROTEIN S2	*Leishmania infantum*	XP_001466537
40S RIBOSOMAL PROTEIN S3	*Encephalitozoon cuniculi*	XP_955676
40S RIBOSOMAL PROTEIN S4	*Mycetophagus quadripustulatus*	CAJ17168
40S RIBOSOMAL PROTEIN SA or P40	*Encephalitozoon cuniculi*	NP_584728
60S RIBOSOMAL PROTEIN L3	*Encephalitozoon cuniculi*	NP_597630
60S RIBOSOMAL PROTEIN L4	*Encephalitozoon cuniculi*	NP_597213
60S RIBOSOMAL PROTEIN L5	*Encephalitozoon cuniculi*	NP_585846
6-PHOSPHOFRUCTOKINASE	*Encephalitozoon cuniculi*	NP_597579
ABC TRANSPORTER (MITOCHONDRIAL TYPE) **#1**	*Encephalitozoon cuniculi*	NP_586426
ABC TRANSPORTER (MITOCHONDRIAL TYPE) **#2**	*Encephalitozoon cuniculi*	NP_586426
ACTIN	*Blakeslea trispora*	AAW32475
ARGININE/SERINE RICH PRE-mRNA SPLICING FACTOR	*Encephalitozoon cuniculi*	NP_597487
ASSOCIATED WITH RAN (NUCLEAR IMPORT/EXPORT) FUNCTION FAMILY MEMBER	*Caenorhabditis elegans*	NP_499369
ATP SYNTHASE	*Encephalitozoon cuniculi*	XP_955732
BELONGS TO THE ABC TRANSPORTER SUPERFAMILY	*Encephalitozoon cuniculi*	NP_597462
cAMP-DEPENDENT PROTEIN KINASE TYPE 1 REGULATORY CHAIN	*Encephalitozoon cuniculi*	NP_597223
CASEIN KINASE 1 HOMOLOG (INVOLVED IN DNA REPAIR	*Encephalitozoon cuniculi*	NP_597600
CATION-TRANSPORTING ATPase	*Encephalitozoon cuniculi*	NP_586078
CHOLINE PHOSPHATE CYTIDYLYLTRANSFERASE	*Encephalitozoon cuniculi*	NP_586276
DNA REPLICATION LICENSING FACTOR MCM2	*Encephalitozoon cuniculi*	NP_584768
DNA REPLICATION LICENSING FACTOR OF THE MCM FAMILY MCM6	*Encephalitozoon cuniculi*	NP_597420
DNA REPLICATION LICENSING FACTOR OF THE MCM FAMILY MCM7	*Encephalitozoon cuniculi*	NP_585977
DNAJ PROTEIN HOMOLOG 2	*Encephalitozoon cuniculi*	NP_586004
DNAK-LIKE PROTEIN	*Encephalitozoon cuniculi*	NP_586489
EUKARYOTIC TRANSLATION INITIATION FACTOR 4A	*Encephalitozoon cuniculi*	XP_955671
FIBRILLARIN (34kDa NUCLEOLAR PROTEIN)	*Encephalitozoon cuniculi*	NP_586197
GENERAL TRANSCRIPTION FACTOR	*Encephalitozoon cuniculi*	NP_597292
GLUCOSAMINE FRUCTOSE-6-PHOSPHATE AMINOTRANSFERASE	*Encephalitozoon cuniculi*	NP_586057
GLYCERALDEHYDE-3-PHOSPHATE DEHYDROGENASE	*Encephalitozoon cuniculi*	NP_586008
GUANINE NUCLEOTIDE BINDING PROTEIN BETA SUBUNIT	*Encephalitozoon cuniculi*	NP_597241
HEAT SHOCK RELATED 70 kDa PROTEIN	*Encephalitozoon cuniculi*	NP_597563
HEAT-SHOCK PROTEIN HSP90 HOMOLOG	*Encephalitozoon cuniculi*	NP_584635
HISTIDYL tRNA SYNTHETASE	*Antonospora locustae*	AAT12372
HISTONE ACETYLTRANSFERASE TYPE B SUBUNIT 2	*Encephalitozoon cuniculi*	NP_586003
HISTONE DEACETYLASE 1	*Encephalitozoon cuniculi*	NP_597645
HISTONE DEACETYLASE	*Encephalitozoon cuniculi*	XP_955621
HISTONE H3	*Mus musculus*	JQ1983
HSP 101 RELATED PROTEIN	*Encephalitozoon cuniculi*	NP_586448
HYPOTHETICAL PROTEIN ECU02_0840	*Encephalitozoon cuniculi*	NP_584609
HYPOTHETICAL PROTEIN ECU02_0950	*Encephalitozoon cuniculi*	NP_584620
HYPOTHETICAL PROTEIN ECU06_0450	*Encephalitozoon cuniculi*	NP_585801
HYPOTHETICAL PROTEIN ECU06_1280	*Encephalitozoon cuniculi*	NP_585884
HYPOTHETICAL PROTEIN ECU07_0530	*Encephalitozoon cuniculi*	NP_585981
HYPOTHETICAL PROTEIN ECU08_1500	*Encephalitozoon cuniculi*	NP_597278
HYPOTHETICAL PROTEIN ECU09_0740	*Encephalitozoon cuniculi*	XP_955628
HYPOTHETICAL PROTEIN ECU09_1700	*Encephalitozoon cuniculi*	XP_955723
HYPOTHETICAL PROTEIN ECU11_1720	*Encephalitozoon cuniculi*	NP_586478
LIM DOMAIN-CONTAINING PROTEIN	*Encephalitozoon cuniculi*	NP_586340
LONG CHAIN FATTY ACID CoA LIGASE	*Encephalitozoon cuniculi*	NP_586206
METHIONINE AMINOPEPTIDASE TYPE 2	*Encephalitozoon cuniculi*	NP_586190
METHIONINE PERMEASE	*Encephalitozoon cuniculi*	NP_585905
NIFS-LIKE PROTEIN (CYSTEINE DESULFURASE) INVOLVED IN IRON-SULFUR CLUSTER SYNTHESIS	*Encephalitozoon cuniculi*	NP_586483
NUCLEAR SER/THR PROTEIN PHOSPHATASE PP1-1 GAMMA CATALYTIC SUBUNIT	*Encephalitozoon cuniculi*	NP_597385
P68-LIKE PROTEIN (DEAD BOX FAMILY OF RNA HELICASES)	*Encephalitozoon cuniculi*	NP_597238
PEPTIDE CHAIN RELEASE FACTOR SUBUNIT 1	*Encephalitozoon cuniculi*	NP_597376
PEPTIDE ELONGATION FACTOR 2	*Glugea plecoglossi*	BAA11470
PHOSPHATIDYLINOSITOL TRANSFER PROTEIN, ALPHA	*Danio rerio*	NP_957229
PHOSPHOMANNOMUTASE	*Encephalitozoon cuniculi*	NP_597365
POLYADENYLATE-BINDING PROTEIN 2	*Encephalitozoon cuniculi*	NP_586226
POLYPROTEIN	*Sorghum bicolor*	AAD27571
PRE-mRNA SPLICING FACTOR	*Encephalitozoon cuniculi*	NP_586183
PROTEIN KINASE B-LIKE PROTEIN	*Plasmodium falciparum*	AAT06260
PROTEIN TRANSPORT PROTEIN SEC23 HOMOLOG (COPII COAT)	*Encephalitozoon cuniculi*	NP_586385
PUTATIVE HYDROLASE-LIKE PROTEIN	*Antonospora locustae*	AAU11090
PUTATIVE ZINC FINGER PROTEIN	*Encephalitozoon cuniculi*	NP_597297
SER/THR PROTEIN PHOSPHATASE 2-A	*Encephalitozoon cuniculi*	NP_584753
SER/THR PROTEIN PHOSPHATASE PP2-A REGULATORY SUBUNIT B	*Encephalitozoon cuniculi*	NP_597423
SERINE/THREONINE PROTEIN KINASE (REQUIRED FOR ACTIN RING AND SEPTATION)	*Encephalitozoon cuniculi*	XP_965898
SIMILAR TO DNAJ-LIKE PROTEIN	*Nasonia vitripennis*	XP_001602403
SIMILARITY TO 14-3-3 PROTEIN 1	*Encephalitozoon cuniculi*	NP_597610
SIMILARITY TO ADP/ATP CARRIER PROTEIN	*Paranosema grylli*	CAI30461
SIMILARITY TO CDC20 (WD-REPEAT PROTEIN)	*Encephalitozoon cuniculi*	NP_597660
SIMILARITY TO Hsp70-RELATED PROTEIN	*Encephalitozoon cuniculi*	NP_584537
SIMILARITY TO HYPOTHETICAL INTEGRAL MEMBRANE PROTEIN YQ55_CAEEL	*Encephalitozoon cuniculi*	NP_597662
SIMILARITY TO HYPOTHETICAL PROTEIN YAAT_BACSU	*Encephalitozoon cuniculi*	NP_597532
SIMILARITY TO HYPOTHETICAL PROTEIN YB36_METJA	*Encephalitozoon cuniculi*	NP_597239
SIMILARITY TO PUTATIVE AMINOACID TRANSPORTER YEU9_yeast	*Encephalitozoon cuniculi*	NP_584803
SIMILARITY TO SKT5 PROTEIN	*Encephalitozoon cuniculi*	NP_586349
SIMILARITY TO TRANSCRIPTION INITIATION FACTOR TFIIA	*Encephalitozoon cuniculi*	NP_597616
STE12 TRANSCRIPTION FACTOR	*Encephalitozoon cuniculi*	NP_586509
STRUCTURE-SPECIFIC RECOGNITION PROTEIN	*Encephalitozoon cuniculi*	NP_586030
T COMPLEX PROTEIN 1 SUBUNIT BETA	*Encephalitozoon cuniculi*	XP_955601
THREONYL tRNA SYNTHETASE **#1**	*Encephalitozoon cuniculi*	NP_586084
THREONYL tRNA SYNTHETASE **#2**	*Encephalitozoon cuniculi*	NP_586084
TRANSLATION ELONGATION FACTOR 1 ALPHA	*Glugea plecoglossi*	BAA12288
TRIOSE PHOSPHATE ISOMERASE	*Encephalitozoon cuniculi*	NP_586329
TUBULIN BETA CHAIN	*Encephalitozoon cuniculi*	NP_597591
U5 ASSOCIATED snRNP	*Encephalitozoon cuniculi*	NP_586393
UNNAMED PROTEIN PRODUCT (Hsp70)	*Candida glabrata*	XP_445544
VACUOLAR ATP SYNTHASE CATALYTIC SUBUNIT A	*Encephalitozoon cuniculi*	NP_586434
VACUOLAR ATP SYNTHASE SUBUNIT B	*Encephalitozoon cuniculi*	NP_586219
ZINC FINGER PROTEIN	*Encephalitozoon cuniculi*	NP_584833

The 97 unique *E. aedis *transcripts which are homologous to genes present in other microsporidia are listed above. Species names and Genbank accession numbers of top BLASTX hits are indicated. Bold text in the "Gene Name" column indicates instances where two different transcripts both had the same top BLASTX hit. Underlining indicates a copy of Hsp70 that is most similar to a protein that remains unnamed in Genbank.

**Table 2:** 

**Gene Name**	**Species Name**	**Genbank Accession Number**
60S RIBOSOMAL PROTEIN L2	*Babesia bovis*	XP_001612300
ADENOSINE KINASE	*Homo sapiens*	AAA97893
HYPOTHETICAL PROTEIN	*Candida albicans*	XP_717148
HYPOTHETICAL PROTEIN PY5484	*Plasmodium yoelii yoelii*	XP_725949
L-ASPARIGINASE	*Dirofilaria immitis*	Q9U518
LYSINE tRNA LIGASE	*Saccharomyces cerevisiae*	CAA39699
PUTATIVE VESICULAR TRANSPORT FACTOR USO1P	*Candida albicans*	XP_710120
SEC63 DOMAIN CONTAINING PROTEIN	*Trichomonas vaginalis*	XP_001580151
PROTEIN PHOSPHATASE 2B	*Cryptosporidium hominis*	XP_666159
WD-40 REPEAT FAMILY PROTEIN	*Arabidopsis thaliana*	NP_201533

The 10 genes which are present in *E. aedis *but absent from other microsporidia are listed above. The species names and Genbank accession numbers of the top BLASTX hit for each gene are also listed.

Approximately a quarter of the transcripts analyzed coded for Hsp70. Almost all of the Hsp70 sequences were most similar to the "heat shock related 70 kDa protein" found in *E. cuniculi *(NP_597563). Single nucleotide variation exists between sequences, usually as 3^rd ^position synonymous substitutions. Where non-synonymous substitutions exist, they are always a single nucleotide and there are no indels between sequences. Mitochondrial-type and DNAK-like Hsp70s were also represented.

**Figure 2:**
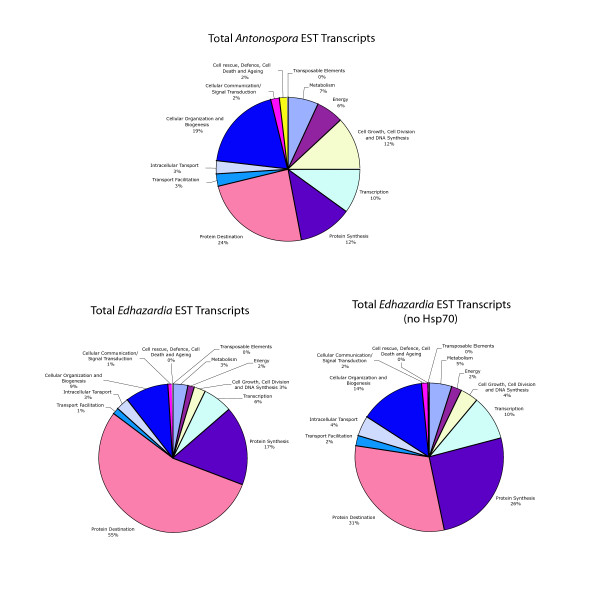
**Total *E. aedis *transcripts represented by COG (Clusters of Orthologous Groups of proteins) category with and without Hsp70.** Total *A. locustae *transcripts are provided for comparison. (*A. locustae *data adapted from Williams et al., 2005.)

**Figure 3:**
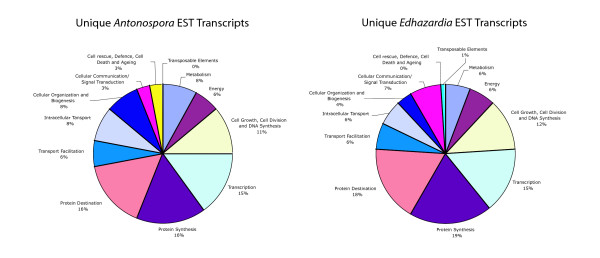
**Unique *E. aedis *transcripts represented by COG category.** Unique *A. locustae *transcripts are provided for comparison. (*A. locustae *data adapted from Williams et al., 2005.)

Genes were assigned to COG categories to allow for comparison with *A. locustae*. Figure 2 illustrates the percentages of total *E. aedis *transcripts that are dedicated to each COG category. Total *A. locustae *transcripts are provided for comparative purposes. As the randomness of the library is uncertain, it is possible that some transcripts are artificially overrepresented. It is therefore more informative to examine unique transcripts (ie. counting multiple transcripts for the same gene only once) rather than total transcripts. Figure 3 displays the percentages of unique *E. aedis *and *A. locustae *transcripts dedicated to each category. Surprisingly, the values are similar and sometimes identical (maximum difference between *E. aedis *and *A. locustae *categories is 5%).

Notable transcripts include a retrotransposon that is similar to LTR retrotransposons present in *Sorghum bicolor *(AAD27571) and *Nosema bombycis *(ABE26655). All belong to the Ty3/Gypsy family of retrotransposons. *E. aedis *also possesses a methionine aminopeptidase 2 gene (MetAP-2), which is present in *E. cuniculi*. There were several transcripts present that appear homologous to proteins found in various eukaryotes, but are absent in other microsporidia examined to date. These include hypothetical or unknown proteins found in *Oryza*, *Danio *and *Plasmodium*, as well as genes encoding proteins with identified functions, such as an adenosine kinase, a lysine-tRNA ligase and an L-asparaginase (See Table 2). In addition, *E. aedis *encodes a putative hydrolase-like protein that is present in *A. locustae*, but absent in *E. cuniculi*.

*E. cuniculi *and *A. locustae *both contain a small number of introns in their genomes and consequently, they have retained a minimal set of splicing machinery. These two organisms are not closely related (See Fig. 1), but they do share a few conserved introns [11]. Therefore, there is reason to suspect that some of these introns may also be present in *E. aedis*. Fortunately, seven transcripts of the gene encoding ribosomal protein L5 (which contains an intron in *E. cuniculi*) were recovered from the *E. aedis *library. These sequences were used to design primers to amplify the L5 gene from genomic DNA. It was found that the *E. aedis *L5 gene does not contain an intron.

### Transcript structure

**Figure 4:**
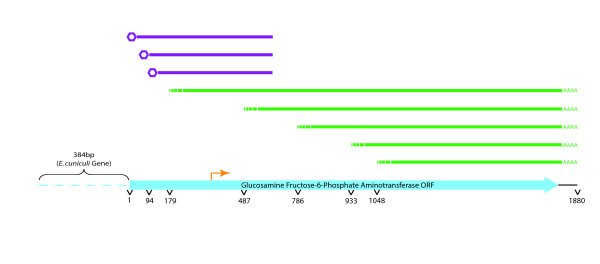
**5' RACE conducted on a moderately represented transcript in *E. aedis *reveals multiple transcription start sites.** ESTs are depicted in green and RACE products in purple. The predicted translational start codon is indicated by the orange arrow. As indicated, the *E. cuniculi *homologue of this gene contains 128 amino acids at N-terminus that appear to be absent in *E. aedis*.

As *E. aedis *is an intracellular parasite and therefore cannot be easily cultured, RNA was limited and the library could not be constructed in a 5' cap-dependent manner. Therefore, nearly all of the inserts encoding the same gene were of different lengths, and most were 5' truncated. However, some of *E. aedis*' transcripts appear to have very long 5' untranslated regions (UTRs) of several hundred base pairs. To further assess transcript structure, cap-dependent 5' RACE (rapid amplification of cDNA ends) was conducted on transcripts from a moderately represented gene, glucosamine fructose-6-phosphate aminotransferase. 5' RACE confirmed that transcript lengths for this gene do vary, with 5' UTRs ranging from 255 to 348 bp (See Fig. 4).

Contrary to the variable start sites of the transcripts, nearly all appear to have identical end sites. The notable exceptions are the heat shock related 70 kDa protein transcripts, which have somewhat variable 3' polyadenylation sites.

There were frequently single nucleotide differences between sequences in contigs, but these differences were usually restricted to silent third position substitutions. In instances where the substitutions are not silent, they are conservative amino acid substitutions. These differences could represent different copies of the same gene or different alleles within the population (UTRs were not available in most cases to determine which).

## Discussion

### Comparing microsporidian transcriptomes

This is the second microsporidian EST project to be conducted and the first from a microsporidian possessing a large genome, allowing for a meaningful comparison of microsporidian spore transcriptomes. Despite the vast differences in genome size and life cycle complexity between *E. aedis *and *A. locustae*, their transcriptomes are highly similar in their compositions. The proportions of unique transcripts encoding proteins devoted to the "protein destination" COG category in both *E. aedis *and *A. locustae *are relatively large (19% and 16%, respectively) (See Fig. 3). It is interesting to note that proteomic work correlates with these results, as the number of proteins in *E. cuniculi *devoted to the "protein destination" COG category form a large percentage of the total proteins present (~28%) that have known functions [12].

When the total number of unique genes found in *E. aedis *and *A. locustae *are compared based on COG category classification, the percentages in each category are close to identical (See Fig. 3). The largest differences lie in the categories of cellular organization and biogenesis, cellular communication and signal transduction and cell rescue, defense, cell death and aging. One notable difference between the two spore transcriptomes is that no transposable elements were recovered in the *A. locustae *ESTs, whereas *E. aedis *transcribes a retrotransposon of the Ty3/gypsy family. Transposable elements have been previously reported to exist in the genomes of *Nosema bombycis *[13], *Spraguea lophii *[14], *Brachiola algerae *and *E. aedis *[8] (See below). To the best of our knowledge, this is the first instance of documented transposable element transcription in microsporidia, and could indicate active transposition.

Nearly 8% of the unique transcripts from *E. aedis *encode genes that are present in various eukaryotes, but are absent from other microsporidia. The existence of these genes has several possible explanations. Sequence data from microsporidia is scarce, and the only completely sequenced genome is that of *E. cuniculi*. Therefore, it is currently impossible to assert that these genes are absent in any microsporidia other than *E. cuniculi*. The possibility exists that they were present in the genome of the microsporidian ancestor, and were lost during genome reduction/compaction events in *E. cuniculi*. These genes could also have arisen from lateral transfer events or they could have come to resemble genes in other organisms by chance or by convergence. Parsimoniously, the first explanation seems most likely, therefore, these data seem to suggest that the ancestor of microsporidia was not, indeed, compact to the extent of *E. cuniculi*.

The MetAP-2 protein is a target for drug therapy in *E. cuniculi *[15]. The *E. aedis *copy of the MetAP-2 gene is very similar to that present in *E. cuniculi*, and contains the amino acid residues that bind the drug fumagillin as well as those believed to coordinate metals. Like *E. cuniculi*, *E. aedis *lacks a polylysine tract at the N-terminus of the MetAP-2 protein that is present in animals, other fungi and plants. This tract plays a role in hindering the phosphorylation of eukaryotic initiation factor 2α (eIF2α), and its absence indicates that the microsporidian proteins likely lack this function [15].

Although our work indicates that the *E. aedis *L5 gene does not contain an intron like its *E. cuniculi *homologue (see Results, above), there is reason to believe that there are introns elsewhere in the genome. There are several transcripts encoding proteins that act in pre-mRNA splicing: an arginine/serine rich pre-mRNA splicing factor (NP_597487 in *E. cuniculi*), a pre-mRNA splicing factor (NP_586183 in *E. cuniculi*) and a U5 associated snRNP (NP_586393 in *E. cuniculi*). These genes comprise 2.2% of the total unique genes found.

### Hsp70

Roughly 28% of total *E. aedis *transcripts encoded some form of Hsp70, a heat shock protein that assists in the folding of other proteins. Hsp70 helps prevent proteins from becoming insoluble and also plays a role in various other intracellular processes, such as apoptosis [16]. The action of Hsp70 allows mutant proteins to continue functioning by being refolded instead of being degraded, which necessitates the costly synthesis of more protein. The number of Hsp70 transcripts in the *E. aedis *ESTs is an order of magnitude higher than was found in *A. locustae *(2%) [17]. We are cautious in this interpretation as we have not quantitatively assessed the transcription level of Hsp70 in *E. aedis*, and it is likely that transcripts of this protein are somewhat overrepresented in the library.

Although no *E. cuniculi *ESTs have been published, Brosson et al. [12] investigated the proteins present in spores. Hsp70 constitutes a moderate amount of all protein present. Brosson and his colleagues classified all proteins based on their COG categories, and found that all "protein destination" proteins together comprise 21% of *E. cuniculi*'s proteome. Intriguingly, Brosson et al.'s [12] experiments indicate that of the four copies of Hsp70 in *E. cuniculi*, the predominately expressed copy of Hsp70 in *E. cuniculi *is homologous to the highly represented transcript in *E. aedis*. In *A. locustae*, the most highly transcribed copy was most similar to the abundantly transcribed copy in *E. aedis *as well [17]. Therefore, it is likely that microsporidia employ similar primary mechanisms to ensure proper folding of proteins.

In other parasites and endosymbionts, such as *Buchnera aphidicola*, Hsp70 is also highly expressed [18] and may constitute up to 10% of the protein contained in the cell at any one time. In species that lead parasitic or endosymbiotic lifestyles, genetic drift and relaxed selection pressure frequently lead to an increased mutation rate. The need for Hsp70 in order for proteins to fold correctly seems to increase with both the size and number of mutations in the protein [16]. Although microsporidian genomes appear to have had little rearrangement, the nucleotide mutation rate seems to be high in this group of organisms [19,20]. Microsporidia could, therefore, contain elevated levels of Hsp70 in order to allow folding of mutant proteins.

### Transposable elements

One of the *E. aedis *ESTs closely matches the integrase domain of the Ty3/gypsy family of retrotransposons. Several of these elements were identified in a GSS of *E. aedis *[8] and a few other microsporidian species, but to the best of our knowledge, this is the first instance in which transcripts of any microsporidian retrotransposon have been found. Transcripts could be indicative of active transposition occurring in *E. aedis*' genome.

Ty3/gypsy retrotransposons exist in many organisms ranging from the microsporidia *Spraguea lophii *[14], *Brachiola algerae *[8], and *Nosema bombycis *[13] to *Saccharomyces*, *Drosophila *and *Sorghum*. Ty3 elements have been well characterized in budding yeast, and exist in 1–4 copies per genome, where they are transcribed by RNA polymerase III. Transcription typically occurs only in haploid cells in the presence of mating pheromones [21]. The *N. bombycis *genome contains at least 8 different retrotransposons in the Ty3/gypsy family, but unlike yeast, they are not exclusively located upstream of tRNAs [13]. Nearly all *N. bombycis *retrotransposons encode a polyprotein containing 5 domains, which exist in a defined order: Gag, protease, reverse transcriptase, RnaseH and integrase. As many of the sequences in the *E. aedis *library appear to be 5' truncated, it is possible that the other domains upstream of the integrase in the polyprotein are also present in genomic DNA. Indeed, the GSS project revealed sequences matching the reverse transcriptase domain [8]. Although the microsporidian *Vittaforma corneae *is also known to possess at least one transposable element [22], it belongs to a different family than those present in *E. aedis *- the L1 family present in humans.

The only completely sequenced microsporidian genome, that of *E. cuniculi *[2], is completely devoid of transposable elements. The existence of similar transposable elements (of the Ty3/gypsy family) in the distantly related *S. lophii, N. bombycis*, *B. algerae *and *E. aedis *(See Fig. 1) implies that this element may have been present in the genome of the ancestor of microsporidia. Therefore, the process of genome compaction that gave rise to the *E. cuniculi *genome likely involved purging transposable elements.

It has been suggested that transposable elements may act to reorganize genes within the genome. Xu et al. [13] compared regions of synteny between *N. bombycis *and *E. cuniculi *chromosomes, as selection appears to be acting to retain gene synteny among microsporidia, even if they are only distantly related [3]. In *N. bombycis*, transposable elements flank these syntenic regions [13]. If *E. aedis' *large genome is partially a product of transposable element proliferation, one would expect much less synteny between this species and other microsporidia. Perhaps future research will elucidate other roles that transposable elements have played in shaping microsporidian genomes, especially since the minute genome of *E. cuniculi *seems to lack them, while they are present in larger genomes.

The functions that these transposable elements perform in a given genome are cryptic at best, but evidence is emerging that they may be more than just simply parasitic DNA. Peaston et al. [23] recently discovered that a class of mouse retrotransposons appears to regulate gene expression in embryos.

### Transcript structure

Transcripts in *A. locustae *typically contain more than one gene. These transcripts do not necessarily contain complete open reading frames for all genes and the genes are frequently in opposite orientations [9]. It is not known how many proteins are made from each transcript or whether this situation is typical for microsporidia, but recent work by Corradi et al. [10] suggests that *E. cuniculi *also possesses multi-gene transcripts.

Unlike *A. locustae *and *E. cuniculi*, *E. aedis *appears to transcribe very few multi-gene transcripts, if any at all. This is not unexpected, given that *E. aedis *genes appear to be separated by large intergenic spaces [8]. The *E. aedis *GSS could not rule out the possibility that local areas of compacted genes might exist [8]. Given the lack of multi-gene transcripts identified, this seems increasingly unlikely.

Also contrary to what is found in *A. locustae*, nearly all of *E. aedis*' transcripts encode proteins in a positive frame (<1% are in a negative frame, compared to 17% in *A. locustae*) [9]. Although antisense transcripts are used in many organisms (possibly also *A. locustae*) to suppress translation, it appears unlikely that this type of regulation occurs in *E. aedis*. Conversely, the large number of antisense transcripts in *A. locustae *may be due to a lack of transcriptional regulation resulting from genome compaction.

*E. aedis*' transcripts seem to start at multiple locations upstream of the start codon (5' UTR length is 180 bp on average) but terminate at the same position with a relatively short 3' UTR (51 bp on average) (See, for example, Figure 4). This is more in line with transcription in *E. cuniculi *and contrasts with the situation in *A. locustae*, where transcripts start directly upstream of the translation initiation site, but often terminate much farther downstream in the adjacent gene [10]. For comparison, the yeast *S. cerevisiae *contains much shorter 5' UTRs than 3' UTRs (15–75 and ~144 bp, respectively [24,25]), a common trend seen in other fungi, plants and animals. The reason for this reversal is unknown, since 3' UTRs are ubiquitously used as translation regulators. It is likely that *E. aedis *lacks some of the translational control mechanisms present in other fungi, plants and animals [26].

## Conclusion

This is the first examination of ESTs from a microsporidian containing a large genome. The extent of genome compaction in the microsporidian ancestor is not known, but the presence of genes in *E. aedis *that have not been found in other microsporidia suggests that extreme reduction and compaction occurred only in specific lineages. Surprisingly, *E. aedis *has a predicted uninucleate spore transcriptome that is highly similar to that of the distantly related *A. locustae*, although the two species have diverse life cycles and genome sizes.

## Methods

Uninucleate *E. aedis *spores were grown and harvested from *A. aegypti *larvae as described previously [27].

*E. aedis *spores were lysed in Ambion's plant RNA isolation aid and lysis/binding solution from an Ambion RNAqueous kit using a bead beater operating at 2500 rpm for 6 minutes with glass beads. RNA was extracted from the resulting supernatant using the RNAqueous kit. A microquantity cDNA library was constructed by Marligen, using the pExpress-1 vector. 1307 clones with an average insert size of 1.5 kb were uni-directionally sequenced using an automated capillary sequencer. Sequences were manually edited and analyzed using Sequencher 4.2 software. Proteins encoded by the transcripts were identified via BLASTX [28] searches performed on the NCBI website (Genbank). Transcripts were identified as encoding a particular protein when BLASTX hits to Genbank proteins had e-values of 10^-4 ^or lower. Transcripts were scored as "present in other microsporidia" when the best BLASTX hit was a gene present in other microsporidia or when the best hit was a gene that has a microsporidian homologue, and the homologue was identified in other microsporidia by BLASTing the *E. aedis *transcript against available microsporidian data. Putative *E. aedis*-specific genes are transcripts that contain open reading frames at least 100 base pairs in size and do not have any BLASTX hits with e-values lower than 10^-3^. In order to facilitate access to the EST sequences, they were uploaded and annotated by the dbEST website [17].

## Authors' contributions

EEG extracted RNA from the *E. aedis *spores, performed and interpreted the sequence analyses and drafted the manuscript. JJB cultivated insect larvae and harvested *E. aedis *spores. NMF conceived of this study, contributed to the interpretation of the results and helped draft the manuscript.

## References

[B1] Wittner M, Weiss LM (1999). The Microsporidia and Microsporidosis.

[B2] Katinka MD, Duprat S, Cornillot E, Metenier G, Thomarat F, Prensier G, Barbe V, Peyretaillade E, Brottier P, Wincker P, Delbac F, El Alaoui H, Peyret P, Saurin W, Gouy M, Weissenbach J, Vivares C (2001). Genome sequence and gene compaction of the eukaryote parasite *Encephalitozoon cuniculi*. Nature.

[B3] Slamovits CH, Fast NM, Law JS, Keeling PJ (2004). Genome compaction and stability in microsporidian intracellular parasites. Curr Biol.

[B4] Streett DA (1994). Analysis of *Nosema locustae *(microsporidia:Nosematidae) chromosomal DNA with pulsed-field gel electrophoresis. J Invertebr Pathol.

[B5] Becnel JJ (1990). *Edhazardia aedis *(microsporidia: Amblyosporidae) as a biological control agent of *Aedes aegypti *(diptera: Culicidae). Proc Vth Int Colloq Invertebr Pathol Microb Control: Adelaide, Australia.

[B6] Becnel JJ, Sprague V, Fukuda T, Hazard EI (1989). Development of *Edhazardia aedis *(kudo, 1930 N. G., N. comb. (microsporidia: Amblyosporidae) in the mosquito *Aedes aegypti *(L.) (DIptera: Culicidae). J Protozool.

[B7] Johnson MA, Becnel JJ, Undeen AH (1997). A new sporulation sequence in *Edhazardia aedis *(microsporidia: Culicosporidae), a parasite of the mosquito *Aedes aegypti *(diptera: Culicidae). J Invertebr Pathol.

[B8] Williams BAP, Lee RCH, Becnel JJ, Weiss LM, Fast NM, Keeling PJ Genome sequence surveys of *Brachiola algerae *and *Edhazardia aedis *reveal microsporidia with low gene densities. BMC Genomics.

[B9] Williams BA, Slamovits CH, Patron NJ, Fast NM, Keeling PJ (2005). A high frequency of overlapping gene expression in compacted eukaryotic genomes. Proc Natl Acad Sci USA.

[B10] Corradi N, Gangaeva A, Keeling PJ Comparative profiling of overlapping transcription in the compacted genomes of microsporidia *Antonospora locustae *and *Encephalitozoon cuniculi*. Genomics.

[B11] Limpright VO, Fast NM personal communication.

[B12] Brosson D, Kuhn L, Delbac F, Garin J, Vivares CP, Texier C (2006). Proteomic analysis of the eukaryotic parasite *Encephalitozoon cuniculi *(microsporidia): A reference map for proteins expressed in late sporogonial stages. Proteomics.

[B13] Xu J, Pan G, Fang L, Li J, Tian X, Li T, Zhou Z, Xiang Z (2006). The varying microsporidian genome: Existence of long-terminal repeat retrotransposon in domesticated silkworm parasite *Nosema bombycis*. Int J Parasitol.

[B14] Hinkle G, Morrison HG, Sogin ML (1997). Genes coding for reverse transcriptase, DNA-directed RNA polymerase, and chitin synthetase from the microsporidian *Spraguea lophii*. Biol Bull.

[B15] Pandrea I, Mittleider D, Brindley PJ, Didier ES, Robertson DL (2005). Phylogenetic relationships of methionine aminopeptidase 2 among *Encephalitozoon *species and genotypes of microsporidia. Mol Biochem Parasit.

[B16] Mayer MP, Bukau B (2005). Hsp70 chaperones: Cellular functions and molecular mechanism. Cell Mol Life Sci.

[B17] O'Brien E, Koski L, Zhang Y, Yang L, Wang E, Gray MW, Burger G, Lang BF (2007). TBestDB: A taxinomically broad database of expressed sequence tags (ESTs). Nucleic Acids Res.

[B18] Wilcox J, Dunbar HE, Wolfinger RD, Moran NA (2003). Consequences of reductive evolution for gene expression in an obligate endosymbiont. Mol Microbiol.

[B19] Thomarat F, Vivares CP, Gouy M (2004). Phylogenetic analysis of the complete genome sequence of *Encephalitozoon cuniculi *supports the fungal origin of microsporidia and reveals a high frequency of fast-evolving genes. J Mol Evol.

[B20] Peer Y Van de, Ben Ali A, Meyer A (2000). Microsporidia: Accumulating molecular evidence that a group of amitochondriate and suspectedly primitive eukaryotes are just curious fungi. Gene.

[B21] Kinsey PT, Sandmeyer SB (1995). Ty3 transposes in mating populations of yeast: A novel transposition assay for Ty3. Genetics.

[B22] Mittleider D, Green LC, Mann VH, Michael SF, Didier ES, Brindley PJ (2002). Sequence survey of the genome of the opportunistic microsporidian pathogen, *Vittaforma corneae*. J Eukaryot Microbiol.

[B23] Peaston AE, Evsikov AV, Graber JH, de Vries WN, Holbrook AE, Solter D, Knowles BB (2004). Retrotransposons regulate host genes in mouse oocytes and preimplantation embryos. Dev Cell.

[B24] Zhang Z, Dietrich FS (2005). Mapping of transcription start sites in *Saccharomyces cerevisiae *using 5' SAGE. Nucleic Acids Res.

[B25] Graber JH, Cantor CR, Mohr SC, Smith TF (1999). Genomic detection of new yeast pre-mRNA 3'-end-processing signals. Nucleic Acids Res.

[B26] Mazumder B, Seshadri V, Fox PL (2003). Translational control by the 3'-UTR: The ends specify the means. Trends Biochem Sci.

[B27] Becnel JJ, Garcia JJ, Johnson MA (1995). *Edhazardia aedis *(microspora: Culicosporidae) effects on the reproductive capacity of *Aedes aegypti *(diptera: Culicidae). J Med Entomol.

[B28] Altschul SF, Madden TL, Schaffer AA, Zhang J, Zhang Z, Miller W, Lipman DJ (1997). Gapped BLAST and PSI-BLAST: A new generation of protein database search programs. Nucleic Acids Res.

